# Coping mechanisms used by caregivers of HIV/AIDS orphans in North West province, South Africa

**DOI:** 10.4102/safp.v66i1.5857

**Published:** 2024-05-14

**Authors:** Boitumelo J. Molato, Salaminah S. Moloko-Phiri, Magdalena P. Koen, Molekodi J. Matsipane

**Affiliations:** 1 NuMIQ Research Focus Area, School of Nursing, Faculty of Health Sciences, North-West University, Mahikeng, South Africa

**Keywords:** caregivers, challenges, caring, coping mechanisms, HIV/AIDS, orphans

## Abstract

**Background:**

Human immunodeficiency virus (HIV) and acquired immunodeficiency syndrome (AIDS) is a pandemic that has affected families and left many children orphaned worldwide. After the death of their parents, HIV/AIDS orphans are often taken care of by caregivers who are faced with overwhelming challenges that affect their capabilities to perform caring tasks. It has been reported that caregivers of HIV/AIDS orphans use different coping mechanisms to deal with the challenges faced during caring. Coping mechanisms play an integral role in maintaining individuals’ physical and mental well-being, particularly those caring for orphans. This study explored coping mechanisms used by caregivers of HIV/AIDS orphans.

**Methods:**

A qualitative design was adopted, and individual semi-structured interviews were used to collect data from 13 caregivers of HIV/AIDS orphans in North West province. Non-probability purposive sampling was used to select the participants. Thematic analysis was used to analyze data. Rigor was maintained throughout the study.

**Results:**

Three main themes were identified with eight subthemes. The first theme includes support from significant others, and subthemes are family support, neighbour support, and life partner support. The second main theme emerged from this study was religious practices and two subthemes namely singing gospel songs and using prayer to cope. The third main theme identified includes the use of social support services, and subthemes were government support, support from local schools, and stokvels and social clubs.

**Conclusion:**

The identified coping mechanisms in this study improved caregiving skills of caregivers to better care for children orphaned by HIV/AIDS.

## Introduction

Globally, South Africa continues to be assailed by the human immunodeficiency virus/acquired immunodeficiency syndrome (HIV/AIDS) pandemic, despite programme interventions to mitigate the spread of the virus. According to literature, South Africa accounts for 20% of new HIV infections worldwide.^1^ By the end of 2021, there were about 38.4 million (33.9–43.8 million) HIV-positive individuals worldwide.^2^ In spite of the initiative of introducing antiretroviral treatment to curb the spread of infection and improve the quality of life among people living with HIV/AIDS (PLWHA), individuals continue to lose lives due to the pandemic. Approximately 40.4 million people globally have lost lives due to HIV/AIDS since the onset in 1981 until 2022.^2^ This pandemic has devastated the world and has resulted in many children requiring care from a variety of people other than their parents. A study conducted in South Africa reported that caregivers of HIV/AIDS orphans are often unable to cope with caring responsibilities due to challenges that they face.^3^ The challenges that caregivers face include lack of financial support to care for HIV/AIDS orphans, and this has dented their caregiving capabilities.^4^

Some caregivers have developed and utilised certain strategies to help them cope with the challenges they face. One of the coping mechanisms that caregivers of HIV/AIDS orphans have employed to counteract the challenges is overconsumption of alcohol.^5^ In the North West province (NWP) of South Africa in the Dr Kenneth Kaunda district, caregivers reported that execution of caring tasks would have not been possible if it was not for the unconditional support they received from the community.^6^ Consistent with the literature, a study conducted in Bojanala District of the NWP in South Africa established that caregivers of HIV/AIDS orphans who were employed received support from their employers.^7^ How caregivers of HIV/AIDS orphans in Ngaka Modiri Molema District of the NWP cope with their challenges remains unknown. Therefore, the researchers in this study identified a need to describe and explore coping mechanisms used by caregivers of HIV/AIDS orphans in this district.

Coping refers to the use of behavioural and cognitive approaches to manage difficulties or threatening situations.^8,9^ Coping mechanisms play an integral role in maintaining the physical and mental well-being of individuals. Once the coping mechanisms are explored, they invariably assist in the formulation of interventions designed to improve the health and well-being of caregivers of HIV/AIDS orphans. Based on the aforementioned discussion, this article sought to describe and explore coping mechanisms used by caregivers of HIV/AIDS orphans in Ngaka Modiri Molema District of the NWP.

## Research methods and design

### Study design

A qualitative, exploratory, descriptive contextual design was used to describe and explore the coping mechanisms used by caregivers of HIV/AIDS orphans.

### Setting

This study was conducted in five municipalities of Ngaka Modiri Molema District of the NWP in South Africa and these include Mahikeng, Ditsobotla, Ratlou, Tswaing and Ramotshere-Moiloa subdistricts. The Ngaka Modiri Molema District of the NWP in South Africa is primarily rural and has high unemployment rates, and it is also home to Mahikeng, the capital of the province. The NWP was rated position 5, with 16.5% by a survey conducted on key HIV indicators, social and behavioural factors, and access to medical interventions in all nine provinces of South Africa among participants of all ages from January 2022 to April 2023.^10^ The Ngaka Modiri Molema is the second largest district in NWP with high prevalence of 6192 orphans aged from 0 to 17 years.^11^ Majority of the society utilise healthcare facilities due to HIV/AIDS epidemic. The services rendered in public health facilities include preventive, promotive, curative and rehabilitative services.

### Study population and sampling strategy

A total of 13 caregivers of children orphaned by HIV/AIDS orphans voluntarily participated in this study. Participated in the study were adults, with ages ranging from 28 to 69 years. Nonprobability purposive sampling was employed to determine eligibility to participate in the study. Only caregivers residing in five local municipalities of Ngaka Modiri Molema and have experience of caring for children orphaned by HIV/AIDS for more than 1 year were eligible to participate in this study (see Table 1 for more details on caregivers’ demographic information). In terms of exclusion, caregivers caring for orphans not orphaned by HIV/AIDS were not considered to participate in the study.

**TABLE 1:** Local municipalities.

Name of the subdistrict	Number of participants
1. Mafikeng	04
2. Ratlou	03
3. Tswaing	02
4. Ditsobotla	02
5. Ramotshere-Moiloa	02

**Total**	**13**

To recruit the caregivers, the recruitment materials that include pamphlets and posters were posted on public places such as churches, schools, community halls, recreational facilities, colleges, shops, et cetera. While both male and female caregivers were targeted to participate in the study, however, only female caregivers showed interest.

Recruitment materials were written in both Setswana and English, as they are the most prominent languages spoken in the areas where data were collected. The information that was written on recruitment materials was the study’s purpose, inclusion requirements, autonomy right, benefits of willingness to participate in the study, and measures to safeguard privacy and anonymity. The contact details of the research assistant and researchers were included in pamphlets and posters so that caregivers who are willing to participate can call to make an appointment. Word-of-mouth recruitment was avoided to avoid indirectly and unintentionally forcing the caregivers to participate in the study. The recruitment procedure was facilitated by the research assistant.

### Data collection

Semi-structured individual interviews and field notes were adopted to achieve the objectives of the study. The interview guide was used to collect data, and it had two sections: one to generate demographic data and the other comprising probing questions. Interviews were conducted at the public health facilities during the day by the main researcher in Setswana because it is the language of communication in the five local municipalities. The reason for collecting data clinical facilities was to avoid stigma and discrimination of the caregivers of HIV/AIDS orphans. The researcher probed participants throughout the study to develop a deep understanding of the phenomenon. Field notes were used to annotate nonverbal cues. Audio recording was used to collect data, and interviews lasted for 45 min – 60 min. The data were transcribed by researchers. Word for word, every single thing said by every participant in an audio recording was typed. Transcribing a single interview took about 2 h – 2.5 h. Each transcript included the name of the research project, the time and date of the interview or recording, the length of the interview, and the order in which the participant and the researcher spoke.

### Trustworthiness

The researcher ensured trustworthiness by adhering to the four principles of trustworthiness, namely, credibility, transferability, confirmability and dependability.^10,12^

### Credibility

Prolonged engagement was applied throughout the study. Interviews were recorded, and field notes were also taken to annotate the verbal and nonverbal cues. The participants in the study were consulted after data analysis for validation purposes.^12^

### Dependability

The qualitative exploratory, descriptive, contextual design methods were used in this study. This principle was adhered to by ensuring detailed data transcription, data coding, data analysis and use of literature control.^12^

### Confirmability

The data were analysed independently by both the researcher and co-coder. After analysing data independently, the two parties compared both themes and subthemes emerging from the study. Open-ended questions were also applied, followed by probing for clarity. The existing literature was used to confirm and equally disprove the study’s findings.^12^

### Transferability

This principle was observed when data saturation was reached, and this was established when participants kept repeating already known information. In this study, data saturation regarding the phenomenon was reached at 13 participants. Purposive sampling was used to select participants eligible for the study, and these resided within the NWP context.^12^

### Data analysis

Six steps of data analysis were adopted in this study to analyse data.^13,14^ The process of data analysis started with reading and re-reading of the transcripts followed by taking of notes. The second step was generating initial codes which entailed arranging data in a comprehensible and methodical manner. The third step was to search for themes; we evaluated the codes and then categorised them into relevant themes. To address the research issue, the codes were arranged into more general themes. After identifying the initial themes in Step 3, we went over, adjusted and expanded upon them as the fourth step. With respect to review themes as the fifth step, its objective is to determine the ‘essence’ of each theme as it has been refined to its final state. Data were sent to the co-coder for coding and consensus regarding the quotes. Themes and subthemes were derived from the data and supported by direct quotations. The codes were grouped into three main themes and subthemes.^13,14^

### Ethical considerations

Ethical clearance to conduct this study was obtained from the North-West University Health Research Ethics Committee (No.: NWU-00196-21-S1). After the study was approved by the relevant institution of higher learning, goodwill letter was written to North West province Department of Health (DoH) to request permission to conduct the study in Ngaka Modiri Molema District. Regarding informed consent, caregivers were given at least 7–14 days to consult and make an informed decision before signing the consent form. The researcher and the research assistant met caregivers who showed interest face-to-face after 14 days to explain further and for signing of the consent form. The consent form was translated to Setswana for caregivers who do not understand English. Moreover, caregivers who were unable to read and write were advised to come along with one person each to act as their witness during the meeting and thereafter sign the consent form as a witness for them. The signing of consent forms took place face to face at the venue chosen by caregivers. Prior to data collection, the purpose of the study was explained to the caregivers and permission to record the interviews was also obtained. The principle of the right to freedom from harm and discomfort was maintained through prevention of physical, emotional and psychological harm of the participants. The participants were informed about their right to voluntarily participate and that they would not be forced to take part in the study. The caregivers were also informed about their right to terminate participation any time when they felt uncomfortable continuing with the study.

## Results

Thirteen semi-structured individual interviews were conducted (see Table 2 for descriptive information of participants).

**TABLE 2:** Description information of participants.

Participants	Age	Gender	Level of education	Number of HIV/AIDS orphans who are cared for by caregivers	Occupation
PA	69	Female	No formal education	1	Pensioner
PB	34	Female	National Senior Certificate	1	Unemployed
PC	56	Female	No formal education	2	Unemployed
PD	44	Female	National Senior Certificate	1	Unemployed
PE	59	Female	No formal education	9	Unemployed
PF	65	Female	No formal education	3	Pensioner
PG	56	Female	No formal education	6	Unemployed
PH	28	Female	National Senior Certificate	1	Unemployed
PI	50	Female	National Senior Certificate	1	Unemployed
PJ	55	Female	No formal education	1	Unemployed
PK	64	Female	No formal education	5	Pensioner
PL	63	Female	No formal education	6	Pensioner
PM	45	Female	No formal education	4	Unemployed

The majority of the participants did not have formal education, while four of them had national certificates. The caregivers were caring for 1–9 orphans. Among the 13 participants, 10 were unemployed and 3 were pensioners.

### Emerging themes

Three main themes and eight subthemes emerged from the analysis of the interview transcripts, summarised in Table 3.

**TABLE 3:** Summary of main themes and subthemes.

Themes	Subthemes
1. Support from significant others	1.1. Family support
	1.2. Neighbour support
	1.3. Life partner support
2. Religious practices	2.1. Singing gospel songs
	2.2. Using prayer to cope
3. Social support services	3.1. Government support
	3.2. Support from local schools
	3.3. Stokvels and social clubs

The first main theme is support from significant others, and subthemes are family support, neighbour support and life partner support. The second main theme is religious practices, and subthemes are singing gospel songs and using prayer to cope. The third main theme is social support services, and subthemes are government support, support from local schools, and stokvels and social clubs.

#### Theme 1: Support from significant others

Support from significant others emerged as the main theme. Life partner support, family support and neighbour support were identified as subthemes.

**Subtheme 1.1: Family support:** Caregivers received financial, emotional and psychological support from family members in this study. However, not all participants received support from family members; only three out of all participants declared family support. The support that they received was not from all family members; some did not bother themselves to usher that support. The participants highlighted sisters, daughters and son-in-laws as individuals who were behind them during hardships. A woman who cares for nine orphans was happy about the support she received from her sister with regard to caring for the HIV/AIDS orphans left under her care. She reported that her sister always assisted her with essentials such as food, toiletry, etc. This was registered as follows:

‘All nine orphans were ill. The first one who passed away was my elder daughter who was followed by her husband three months later. They both left five children as HIV/AIDS orphans. My youngest daughter was also sick and passed away leaving behind one child as an orphan. Other orphans belong to my other sister. My sister never disappoints me whenever I need her assistance. She is caring, and very supportive. Most of the time she assists me with the basic needs such as groceries.’ (PE, 59 years)

Another participant caring for six orphans who live with HIV reported that she received money monthly from her daughter’s family to beef up the grocery. She expressed herself as follows:

‘My second and third born passed on in 2019 and 2020 respectively, leaving their children behind as orphans. The second one left behind four children whilst the third one left behind two children. These orphans are attending school; one is in grade seven, two are in grade three, another one will be starting school next year, and the last one will be starting pre-school next year. My daughter, together with her husband, always deposit cash for us so that we can buy essentials like grocery, toiletry, and other stuff that we may need.’ (PL, 63 years)

A woman caring for three HIV/AIDS orphans reported that she relied on her younger sister when their grocery finishes. The following vignette captures her submission:

‘My younger sister is always on our side when we are going through difficult times. She assisted us with groceries when we are in need.’ (PF, 65 years)

**Subtheme 1.2: Neighbours support:** Some of the participants were so grateful for the physical support that their neighbours offered them. A woman who is in care of two HIV/AIDS orphans reported that her neighbour assisted her usually when they were low on basic needs, particularly food. This was expressed as follows:

‘Our neighbours are always supportive during difficult times; I approached them to seek for assistance depending on the type of food I will be lacking of in the household. In the villages we don’t struggle a lot because we live like one family with neighbours through thick and thin.’ (PC, 56 years)

The same sentiment was shared by another participant caring for one HIV/AIDS orphan. She said:

‘Our neighbours are extremely supportive. They play an important role in our lives. There is a Setswana idiom that states that “Matlo go sha a a mabapi” translated to mean “neighbours are bound to help each other in times of need” and this is really working for us. When I need basic needs like maize meal or sugar, she supported us without any complaints. Even when she is in need of anything that she doesn’t have, she came to us to seek for assistance.’ (PI, 50 years)

**Subtheme 1.3: Life partner support:** The participants who were having life partners were thankful for their support during difficult times of caring for HIV/AIDS orphans. In this study, life partner refers to two individuals who are not married but committed to intimate relationship. The participants appreciated their fiancés for being available when they were unable to provide the families with food and other essentials that are needed on day-to-day care. A woman taking care of an orphan who is an intellectually disabled daughter hailed her partner who is lending a helping hand to them unconditionally. This was stated in the following vignette:

‘My baby daddy brought us food when our grocery is finished. He is doing odd jobs but he never forgets us when he gets paid. He was here yesterday to give us food. I so wish that God can bless him with [*a*] full time job so that he can take care of us full time.’ (PD, 45 years)

Another participant was fortunate to have a caring, loving and selfless partner who supports them through his income from the spaza shop that he owns. She said:

‘My boyfriend supported us with everything that we need. He is owning a spaza shop though it’s not so busy because Pakistanis are all over the village and our people support them compared to South Africans. Regardless of the circumstances, he is always available to assist us with the little that he got from his spaza shop.’ (PB, 34 years)

A woman caring for nine orphans was happy that her husband supports them with his pension money to buy groceries for the household. The following captures the sentiments on caring:

‘My partner is a pensioner and he is very supportive. We are trying to use the little money that he receives to access groceries. Though it is not enough but at least the burden is not too much. I combine the foster child grant and my partner’s pension to buy grocery that can last us for at least the whole month.’ (PE, 59 years)

#### Theme 2: Religious practices

Religious practices emerged as the second theme and the following two subthemes were identified, namely, singing of gospel songs and using prayer for coping.

**Subtheme 2.1: Singing of gospel songs:** The participants praised religious practices for the significant role that it plays in easing challenges that emerge from execution of caring responsibilities. The testimony was shared by a woman who cares for six orphans and reported that gospel songs make her feel relieved and strengthened to face the challenges head-on. The following was echoed:

‘I played religious hymns on my radio to calm down my emotions. Playing hymns on radio gave me peace of mind. The hymns are medicine to me when I come across the challenges of caring for this orphan. I am not a good singer but I do have a favourite hymn in the hymn book “Ke tsamaisiwa ke morena o gaufi le nna o a ntisa mo ditekong tsotlhe tsa lefatshe. O nkgoga ka letsogo la gage le le thata” [*Translated it means “Almighty God protects me throughout the journey of life. He strengthens me in all life tribulations and always extend his helping hand*.”] I don’t mind playing this hymn repeatedly the whole day it relieves me and it makes me feel better.’ (PG, 56 years)

The participants used gospel songs in different ways in order for them to cope with stressful situations. A woman who takes care of one orphan reported that opening a hymn book and reading the message when overwhelmed by caring challenges is therapeutic. This is what she said:

‘I rely on hymns when I am hurt when the challenges are too heavy for me to handle. I am not gifted in singing but there are hymns that calm me down when lot of things are happening and I don’t have solutions to those problems. Although there are hymns that make me feel like crying, but when things are tough, I become a better person when I listen to the hymns.’ (PA, 69 years)

**Subtheme 2.2: Using prayer for coping:** The participants highlighted prayer as a weapon that they use to cope with all the challenges they face in caring for HIV/AIDS orphans. One of the participants caring for three orphans reported that prayer strengthens her to tackle all obstacles in her way of caring for these orphans. She expressed herself as follows:

‘Sometimes when I am overburdened by the challenges I just kneel down and pray with all my heart. Prayers do wonders. You can get everything you want as long as you believe and pray with faith.’ (PK, 59 years)

The significance of prayer was pointed out by another participant caring for one orphan. The following was echoed:

‘Prayer is so important. When I pray for something, God never disappoints me. I always get something in return and I trust him when it is tough.’ (PI, 50 years)

#### Theme 3: Social support services

Social support services emerged as a third theme and the subthemes identified include government support, support from the local clinics, stokvels and social clubs. These are discussed in detail in the subsequent segments.

**Subtheme 3.1: Government support:** The participants verbalised that the social grants provided to them by the government assisted them a lot to buy groceries for the household and make savings for emergencies that may arise from the difficulties they experience in caring for HIV/AIDS orphans. A woman caring for two orphans expressed herself as follows:

‘Government is taking care of us by ensuring that HIV/AIDS orphans receive foster care grants. I use the money to buy grocery and other important items for the house. I also save certain amount of the social grants in case one of the orphans becomes ill.’ (PC, 56 years)

Another woman caring for six orphans verbalised that they are monitored by social workers to check if they are not using money fruitlessly. She said:

‘The money that we receive from government is not enough to buy stationery, uniform, grocery and clothes for orphans. All of six HIV/AIDS orphans I care for receive foster grants. We are bound by law to save a certain amount monthly. It is not a matter of we spend the whole money on grocery, clothes, and stationery. I have opened bank accounts for six of them and I account to the social worker. She likes to pay us unannounced visits to see if the orphans are well taken care of.’ (PL, 63 years)

A pensioner who cares for five HIV/AIDS echoed the importance of the social grants. She said:

‘We rely on foster care grant and my pension to buy groceries, winter, and Christmas clothes for them. My biggest problem is [*that the*] money that we are receiving from government is not enough to meet the needs of [*these*] orphans. When the children grow up, they need new clothes and other necessary items. I always run short of money to buy them clothes that fit them according to age and weight. Only two of the HIV/AIDS orphans I care for are registered for foster care grants, and three of them are over the age of 18 and amongst five orphans there is a girl who is older … she will be completing matric this year.’ (PK, 64 years)

**Subtheme 3.2: Support from the local schools:** Some of the participants who are unemployed and do not receive social grants are assisted by local schools. They receive basic items that they need for daily living, such as clothes and food parcels. A woman caring for one orphan echoed the following:

‘I am not getting any support except from the primary school in this village. They always provide us with groceries because of their understanding that we are a needy family. The school does not provide us with grocery hampers every month, but at least the little that we receive from them helps us not to go to bed hungry and we appreciate it.’ (PH, 28 years)

A neighbour who decided to take over caring responsibilities from a blind woman caring for six HIV/AIDS orphans hinted that some of the teachers ensure that orphaned children are not deprived of their rights to attend school by making sure that they pay for their fees at school. The participant spoke in the following words:

‘The caregiver of these orphans does not have good vision; she is blind. She is receiving pension grant and it is not enough to cater for their basic needs i.e., children’s school and foody. She is struggling … she does not have clothes; the money is not enough. I am trying my level best to distribute this money equally so that they can pay school fees, buy grocery, toiletry and washing soap. This lady is staying with seven children, as a neighbour I am supporting them because the caregiver is blind. For stationery, we depend on the school teachers at the school where these are attending are … they are so helpful. They sometimes buy toiletry, grocery, and take responsibility to pay school fees.’ (PG, 56 years)

The same sentiment was shared by another participant who reported that:

‘The orphan under my care is not receiving social grants because of age. He is 19 years old. The aunt is not supportive to this orphan, she never bothers herself to at least meet us halfway by buying him stationery, school uniform, and clothes in the least. The school sometimes assists him with stationery and other learning materials though it is not regularly.’ (PI, 50 years)

**Subtheme 3.3: Stokvels and social clubs:** A woman who cares for one orphan joined stokvels and social clubs to cope with day-to-day caring responsibilities. At the end of the year, they receive groceries which last more than a month. She expressed herself as follows:

‘I survive by making plans like now I have joined a stokvel because social grants alone are not enough to cover the needs of [*the*] children that we are caring for. Everything is expensive so stokvels are very helpful. On [*a*] monthly basis I am expected to pay R250 from January to December. The groceries that we receive from stokvel are [*a*] bit [*of*] a relief to us. These groceries help us a lot because we are able to assist neighbours and other people in the village who are always on our side when we are faced with tough situations.’ (PB, 34 years)

The importance of stokvels was also raised by a participant who cares for one orphan and she hailed stokvels as helpful and assists her to deal with catering to the demands of the HIV/AIDS orphan. The following was raised by the participant:

‘We are unemployed, we cannot afford to live like other families who get everything at any time they wish. The orphan that I am taking care of is my friend’s son and his mother passed away two years back. He asked me to come and stay at his place because things got worse after the death of his mother. His family is not treating him well and they don’t care about him. The size of my family has increased and the demands are high. The stokvel that I have affiliated with stokvels and I contribute R300 monthly. It is the stokvel for groceries. We share groceries at the end of the year so we don’t struggle a lot during [*the*] festive season with food and other significant essentials as they are catered for in grocery hampers.’ (PI, 50 years)

## Discussion

The objective of this study was to describe and explore the coping mechanisms used by caregivers of HIV/AIDS orphans in Ngaka Modiri Molema District, NWP of South Africa. This study identified different coping mechanisms that caregivers of HIV/AIDS orphans used to cope with challenges. Our results show that caregivers used problem-focused and emotion-focused coping mechanisms to deal with day-to-day challenges they faced in caring for HIV/AIDS orphans.^15^ When it comes to problem-focused coping, the person exhibits both cool, collected, purposeful attempts to solve the problem and aggressive interpersonal attempts to change the circumstances.^16^ Previous studies mostly defined problem-focused coping as the mechanisms that individuals employ to lessen stressors.^17^ The problem-focused coping mechanisms used by caregivers in this study include support from family, neighbours, life partners, government, local schools, stokvels and social clubs. Individuals who are experiencing emotion-focused coping exhibit the following indications and symptoms: admitting responsibility, distancing themselves, self-controlling, seeking out social support, avoiding situations that could lead to trouble and positive reappraisal.^16^ With respect to emotion-focused coping, researchers described it as the mechanisms that are used by individuals to reduce the effect of emotional distress.^18^ The examples of emotion-focused coping mechanisms used by caregivers were singing gospel songs and use of prayer to cope. Although the mechanisms provided caregivers with temporary solutions to the problem, they were unable to deal with the aftermath of the stress caused by the challenges.

Findings of this study mirror a considerable amount of literature that has been published on how caregivers used government and spiritual to cope with caring challenges in Southern African Development Community (SADC) countries.^19^ Consistently with existing literature, caregivers in Limpopo province, South Africa, depended on foster care grants provided by government for survival.^20^ In a similar vein, the study conducted in Bulilima District, Matabeleland South province, Zimbabwe, stated that the government of Zimbabwe supports caregivers of HIV/AIDS orphans by providing 50 kg of grain every month and 10 kg of rice as a complementary gift from the president.^21^ In contrast to earlier findings, government and its partners failed to provide caregivers of HIV/AIDS orphans at Gutu District of Zimbabwe with food parcels.^22^ This finding supports previous studies that revealed that systems put in place by different states to support caregivers of HIV/AIDS orphans are not adequate, effective and efficient. In this study, caregivers mentioned government as the only source of support that did not mention any stakeholders or nongovernmental organisations. Therefore, this study supports previous studies’ recommendations, which state that caregivers should receive training in business, finance and vocational skills as part of an efficient programme to enhance home finances and help families become financially independent.^23^ This can only be achieved if government collaborates with nongovernmental organisations to roll out the suggested programmes to develop caregivers of HIV/AIDS orphans.

It seems like life partner support found by this study is a new contribution to the body of knowledge based on the fact that there is dearth in literature to support this finding. The available literature provides empirical evidence of a different setting which is a youth care centre whereby a woman was working as a caregiver of HIV/AIDS orphans.^24^ Her husband was her shoulder to cry on every time when she got knocked off and had been overwhelmed by the stressful events from the working environment.^25^ On that note, researchers deemed that this literature is not relevant to support this finding for the fact that there is a variation between the two contexts of caring for HIV/AIDS orphans. Therefore, this study suggests that more research should be conducted to explore the significance of life partner support on caregivers caring for HIV/AIDS orphans.

Significant support to others is an important theme to this study because having a strong support system develops individuals to deal with problems more independently and be more resilient, as it fosters independence, self-assurance and self-worth.^26^ A support system is a network of people who are always willing to lend a hand when necessary to keep you going through difficult times.^26^ Within the context of this study, caregivers are discharging caregiving responsibilities in an environment that is not conducive. They relied on psychological, financial and emotional support from members of the family to overcome challenges that comes in their line of caring. Findings of this study support what were reported on the studies conducted in South Africa and Swaziland that revealed that majority of caregivers who were not coping depended on one or two family members for physical and emotional support.^25,26^ Caregivers require family members who are honest and trustworthy so that they can be transparent to share all difficulties that they encounter in caring for children orphaned by HIV/AIDS.

If family members become aware of the stressful situations that caregivers are exposed to, they can easily show humanity by being part of that adversity by lending a helping hand and even provide psychological and emotional support by owning the situation.^27^ By doing so, caregivers will not be able to feel the burden of caring due to the support that they receive from family members. In support of this statement, the study conducted by Osafo and other researchers reported that caregivers with good support system were coping well with caring responsibilities.^28^ Additionally, effective support systems according to some researchers provide people with psychological support to cope with stressful situations and frequently provide them with the fortitude to persevere and even prosper.^29^ People feel more capable of handling life’s stresses when they are surrounded by kind and encouraging people who are always available for emotional and psychological support. This study further illustrated that neighbours created a conducive environment because they often share their groceries with some of the caregivers of HIV/AIDS orphans and this exhibits ubuntu where there is kindness, selflessness and a human connectedness to the others who find themselves in this predicament. Ubuntu means ‘umuntu ngumuntu ngabantu’, suggesting I am a person through other persons. This adage communicates a fundamental regard and concern for other people.^30,31^ To support this notion, a phenomenological study conducted in Ghana established that neighbours were always available whenever the caregivers of HIV/AIDS orphans failed to cope with caring demands and needed someone with whom to communicate.^32^ The neighbours showed ubuntu by offering the caregivers of HIV/AIDS orphans emotional support when they were trapped in a difficult situation.^33^ Thus far, evidence from the current study clearly verified that neighbours did not turn their back against caregivers of HIV/AIDS orphans during difficult times. The availability and willingness of neighbours played an essential role in day-to-day activities to support caregivers in rendering care to HIV/AIDS orphans. The results of this study confirmed that neighbours were the only source of support system within the community.

Our findings also show that caregivers sang gospel songs and used prayer as coping mechanisms to address the challenges they experience when caring for HIV/AIDS orphans. This activity benefitted caregivers in two different ways. For example, some of the caregivers preferred to open a hymn book and interpreted lyrics of the gospel songs to heal emotions when they cannot cope. These findings are consistent with the study conducted in Zimbabwe that reported that caregivers sang gospel songs to comfort themselves.^22^ Likewise, caregivers sang gospel songs to draw strength from God so that they can effectively deal with challenges.^5,34^ Singing and listening to gospel songs strengthened the momentum of the caregivers to better care for HIV/AIDS orphans and alleviate caregiving burden. Furthermore, the study findings verified that the caregivers of HIV/AIDS orphans used prayer to cope with the stress they experienced during caregiving. Similar results were reported by the study conducted in Uganda whereby caregivers used prayers to communicate their challenges and demands to God.^21,27^ Moreover, caregivers relied on spiritual support by putting God first in everything to avert caregiving burden.^28,34^

Religious practices is described in the literature as an emotion-focused coping mechanism people adopt to manage emotions brought on by stressful circumstances.^29,35^ The decisions of caregivers for employing religious practices were based on the transactional theory that asserts that coping is an evolving process that assists individuals to adapt to the circumstances of handling various internal and external demands.^25,31^ Moreover, previous studies showed how religious practices and coping mechanisms are related.^30,31,32,36,37,38^ These researchers further contend that religious practices are crucial to coping strategies because they seem to be the first line of defence against suffering and discomfort. People who are very spiritual may find it easier to use a variety of religious coping mechanisms under pressure (such as prayer, meditation and religious assessments).^30,31,32,36,37,38^ Scientific evidence in this study revealed that caregivers benefitted from using prayer and singing of gospel songs as a weapon to battle all difficulties that interfered with their abilities to discharge effective and efficient caregiving HIV/AIDS orphans. However, it is not always the case that religious practices are the only hope that can be relied upon to overcome the challenges. Literature revealed that some of the caregivers in the study conducted in Bulilima District in Zimbabwe had faith in ancestors making execution of caring duties easy and beneficial to the orphans.^15,20^ Data reported by this study and existing literature appear to support the assumption that ethnicity plays a pivotal role in the lives of individuals, although there is scarcity of available literature to support this statement.

This study also revealed that educators from local schools provided caregivers of HIV/AIDS orphans with some support in the form of school fees, stationery and grocery hampers. The support was in a form of donation whereby educators contributed a certain amount of money to buy such items for caregivers. This finding is consistent with the results of the study conducted in Western Cape province of South Africa that showed that educators supported HIV/AIDS orphans with school uniform in dire circumstances.^33,39^ In a study conducted in Kenya, it was confirmed that educators and the governing bodies at schools supported interventions for orphaned adolescents which included paying elementary and secondary schools’ fees, uniform expenses and other school activities.^34,40^ The support they received from different schools enabled HIV/AIDS orphans to focus on their studies without dropping out from school. On the other hand, it was a relief on the caregivers of HIV/AIDS orphans because the support from local schools made caring responsibilities executable.

Findings of the study further established that caregivers of HIV/AIDS orphans joined different stokvels and social clubs to cope with the caring challenges they face. Stokvel is an informal self-help group that draws individuals together with the goal of contributing money on a weekly or monthly basis to meet a need that arises from poverty, unemployment and financial instability.^35,41^ In this study, the caregivers reported that they affiliated with different stokvels and social clubs and contributed monthly to the financial viability of these stokvels. The money is usually shared at the end of the year to purchase groceries in bulk for equal distribution among all members. In support of this finding, the study conducted in Kenya verified that caregivers appreciated stokvels and social clubs for supporting them in caring for HIV/AIDS orphans.^13,18^ The authors further reported that caregivers borrowed money from stokvels and social clubs to start individual small businesses. The initiative assisted caregivers to handle non-material pressure, for example, burden of caring, emotional strain pressure emerging from execution of caregiving HIV/AIDS orphans. The researchers are of the view that social clubs played a significant role in the psychological and emotional well-being of the caregivers. Literature has highlighted the value of social club membership as a means of enhancing lives via the development of new connections.^36,42^ Therefore, social clubs and stokvels are generally assumed to play a significant role in creating a supportive environment for caregivers.

### Limitations

It was not easy for researchers to get to data collection sites based on the fact that Ngaka Modiri Molema District is scattered. The researchers had to travel as far as more than 100 km to get hold of the caregivers. Some of the caregivers cancelled the appointments at the 11th hour and led to re-scheduling another day for the interview.

## Conclusion

Regardless of the study limitations, the results of this study support previous studies that were conducted about the phenomenon yet bring new contribution that calls for more studies to be conducted about it. Partner support was identified as a new contribution; this study further revealed that partners were available to provide financial, psychological and emotional support. Furthermore, this study sheds more light on problem-focused and emotion-focused coping mechanisms. The caregivers of HIV/AIDS orphans used adaptive coping mechanisms to tackle their difficulties that arose from caring tasks. Focus of caregivers was mainly on addressing challenges that are related to a lack of food and other essentials.

### Recommendations

Based on the findings of this study, the researcher recommends that government should offer caregivers caring for orphaned children living with disability care dependency grant. This is because they cannot look for employment reason being that orphaned children whom they are caring for require special care. Government lacks the capacity to support the caregivers to the level of their satisfaction; therefore, they should consider to forge partnership with other stakeholders to capacitate caregivers of HIV/AIDS with skills that would assist them to be dependent and stimulate them to initiate income-generating projects that are sustainable.
